# Trust and verification in AI-enabled physician chatbots for chronic disease management: evidence from digital health behavior

**DOI:** 10.3389/fmed.2026.1830356

**Published:** 2026-06-24

**Authors:** Haitham Alzghaibi

**Affiliations:** Department of Health Informatics, College of Applied Medical Sciences, Qassim University, Buraydah, Saudi Arabia

**Keywords:** AI physician chatbots, chronic disease management, digital health verification, health information seeking, mHealth platforms

## Abstract

**Background:**

Advances in digital health technologies have transformed how individuals with chronic diseases seek and use health information. Patients increasingly rely on online sources, including search engines, social media, and messaging applications, to understand symptoms and manage chronic conditions. However, these digital environments can also expose users to misinformation or conflicting advice. Artificial intelligence (AI) enabled tools and mobile health (mHealth) services have emerged to assist patients in identifying symptoms, verifying health information, and supporting timely health decisions. Despite these developments, limited conceptual work has examined how individuals with chronic diseases integrate such tools into their health information–seeking and decision-making processes.

**Objective:**

This study aimed to develop and empirically illustrate a model explaining how individuals with chronic diseases seek and verify digital health information using AI-enabled tools and how these processes influence trust and health-related decision-making.

**Methods:**

A cross-sectional survey was conducted among adults aged ≥ 18 years diagnosed with diabetes or hypertension. Participants were recruited through chronic disease support groups on Facebook and WhatsApp. The survey assessed digital health information–seeking behavior, verification practices, trust in AI-enabled physician chatbots and national mHealth services, and their role in health-related decision-making. Descriptive statistics and visualization analyses were conducted using R.

**Results:**

Health information seeking occurred across multiple digital platforms, with considerable overlap between messaging applications, social media, and web-based sources. Participants reported using AI physician chatbots and national mHealth services mainly to verify health information encountered online. Trust in AI diagnostic support tools was moderate, indicating cautious but active engagement. Most participants used these tools to support clinical consultations rather than replace professional medical advice.

**Conclusion:**

Verification behavior and trust play key roles in how individuals with chronic diseases engage with digital health information. AI-enabled mHealth tools may function as complementary decision-support resources that help patients verify information and interpret symptoms while supporting informed health decisions alongside traditional healthcare services.

## Introduction

Over the past few years, more attention has been paid to how patients look for health information beyond traditional healthcare settings (1–4). The ease of access to smartphones has made online health content a part of everyday life (5). Many patients use digital sources to make routine health decisions (3). However, the reliability of this information is unclear. Previous research has indicated that people living with chronic conditions are especially active in searching for health information (6). Particularly, patients with diabetes and hypertension often require ongoing advice to help manage their condition over time. Previous studies have shown that patients are frequently exposed to health messages shared on social media and messaging applications. While some information may be helpful, other content may be inaccurate or misleading (6).

Evidence indicates that exposure to unverified health information can influence patient behavior and decision-making. In particular, difficulties assessing credibility may lead to confusion or inappropriate self-diagnosis. As noted in earlier research, patients do not always possess the skills required to critically evaluate online health content (3). Therefore, the need for reliable verification tools has become more apparent.

In response to these challenges, mobile health applications have been promoted as a means of supporting patients beyond clinical encounters. Several studies have reported that mHealth tools can support monitoring, education, and communication (1). However, these findings are mixed. In particular, little attention has been paid to how patients use such platforms to verify health information encountered elsewhere (3).

Recently, artificial-intelligence-based physician chatbots have been introduced into mHealth platforms. In this study, the term “AI physician chatbot” refers specifically to generative AI conversational agents based on large language models (e.g., ChatGPT, Gemini, Microsoft Copilot, and similar dialogue-based assistants) that produce free-text, contextual responses to patient queries about symptoms, medications, and chronic disease management, rather than to traditional rule-based or decision-tree symptom checkers (e.g., WebMD Symptom Checker, Isabel, Ada Health’s structured triage flow) that match user inputs to pre-coded diagnostic pathways. This distinction is important because the verification, trust, and reliance behaviors examined in the DHVAT framework differ meaningfully between the two categories of tools. Existing research suggests that these tools may help patients interpret symptoms and clarify uncertainty (6, 7). However, concerns regarding trust and perceived reliability remain. Moreover, most studies have focused on technology acceptance rather than behavioral pathways (7).

In Saudi Arabia, national digital health initiatives, including the Sehhaty app, reflect a broader shift toward digitally enabled care. Despite this increased uptake, limited conceptual work has explained how patients move from information exposure to verification and decision-making (8). Therefore, this study addresses this gap by developing a conceptual framework that provides an empirical description of digital health information-seeking behavior, verification behavior associated with health information obtained from digital sources, and decision-making about health among individuals with chronic diseases, specifically those with diabetes and hypertension. Focusing on patients with diabetes and hypertension allows investigation of individuals with chronic diseases who are actively engaged in ongoing disease management and are therefore more likely to interact with digital health technologies. In addition, this study contributes to the literature on digital health behavior by showing how AI physician chatbots and national mobile health applications may support information verification and decision-making among individuals with chronic diseases in an increasingly complex digital health ecosystem, rather than replacing physician-provided medical care.

The Digital Health Verification and AI Trust (DHVAT) framework developed in this study draws on three complementary theoretical perspectives: Wilson’s Model of Information Behavior, the Technology Acceptance Model (TAM) (9, 10), and the Unified Theory of Acceptance and Use of Technology (UTAUT2) (11). Wilson’s model provides a conceptual basis for understanding health information-seeking behavior as a need-driven process, in which individuals actively consult multiple sources to reduce uncertainty about their health conditions (9). In contemporary digital environments, this process frequently involves exposure to health information across several platforms, including social media, messaging applications, and web-based resources (12).

Building on this foundation, the TAM offers insights into how individuals evaluate emerging digital health technologies (12, 13). In particular, perceptions of usefulness and ease of use influence whether patients adopt tools, such as AI physician chatbots, to assist in interpreting symptoms, managing chronic conditions, and verifying health information online. The constructs examined in this study–diagnostic support, self-management support, information verification utility, and AI chatbot trust reflect these evaluation processes.

**FIGURE 1:**
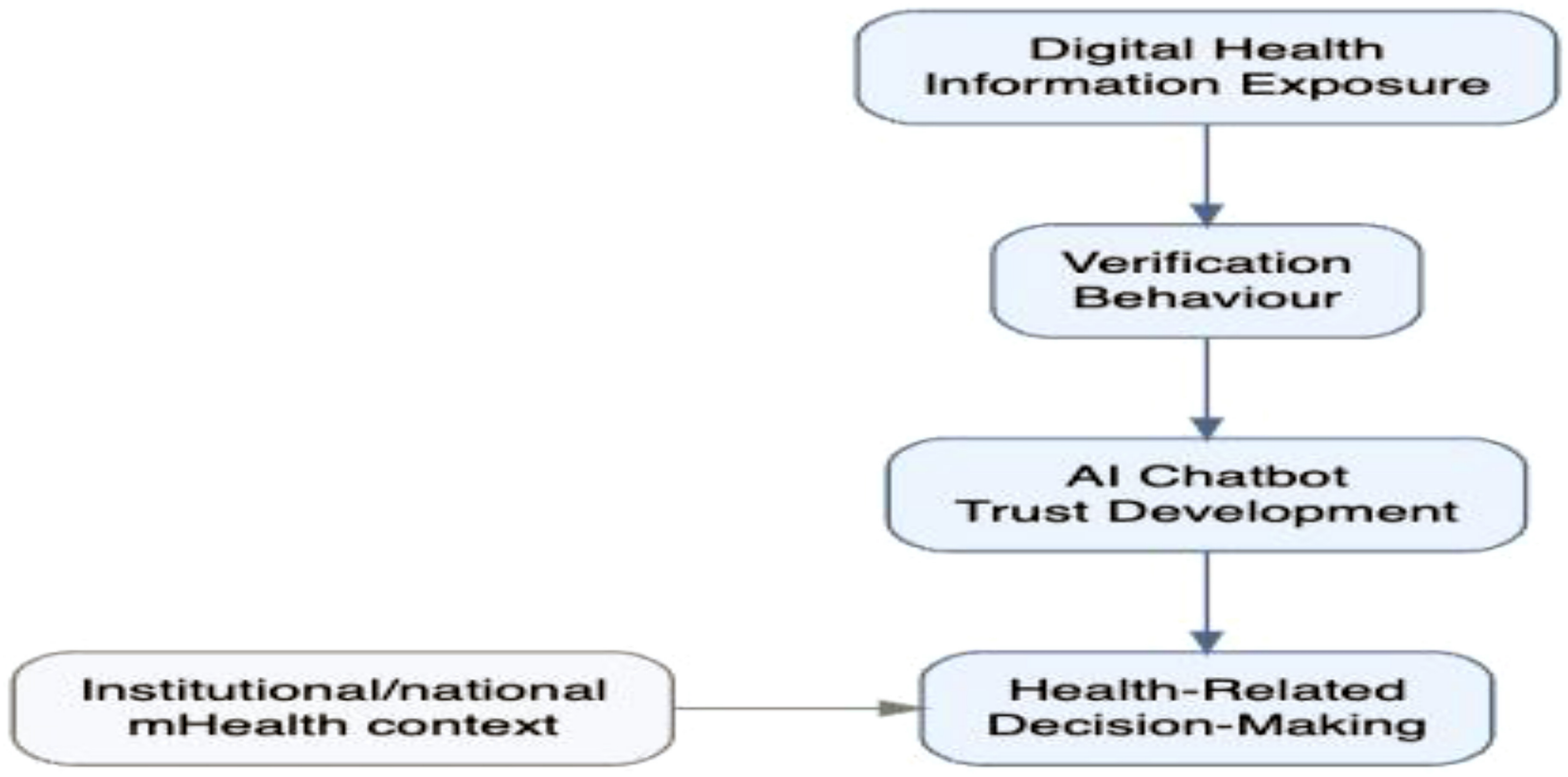
Conceptual framework of the digital health verification and AI trust (DHVAT) model linking information exposure, verification behavior, AI chatbot trust development, and health-related decision-making.

The UTAUT2 further highlights the role of social and contextual influences on technology use. Drawing on these perspectives, the DHVAT framework conceptualizes digital health behavior as a four-stage process: information exposure, verification behavior, AI chatbot trust development, and health-related decision making. The framework emphasizes verification behavior as an intermediary stage, illustrating how patients evaluate digital health information before incorporating AI-enabled guidance into health decisions (see Figure 1).

In addition to integrating the established models, the DHVAT framework makes three distinct theoretical contributions. First, it positions verification behavior as a discrete intermediary construct between digital information exposure and AI-enabled decision support, addressing a gap in TAM and UTAUT2, which treat acceptance and use as a direct outcome of utility and contextual perceptions and do not explicitly model the credibility-checking processes that occur in misinformation-rich environments. Second, the framework reconceptualizes trust in AI not as an antecedent of acceptance (as in conventional TAM extensions) but as a downstream construct shaped by patients’ verification experiences, reflecting the iterative nature of trust formation in chronic disease self-management. Third, by embedding Wilson’s information-seeking lens within a technology acceptance scaffold, the framework explicitly accommodates multiplatform exposure as the starting point of digital health behavior, rather than assuming a single technology of interest. Accordingly, the following hypotheses were tested in the empirical model:

*H1:* Perceived diagnostic support from AI physician chatbots is positively associated with perceived self-management support.

*H2:* Perceived diagnostic support from AI physician chatbots is positively associated with information verification utility.

*H3:* Perceived self-management support is positively associated with trust in AI physician–chatbot guidance.

*H4:* Information verification utility is positively associated with trust in AI physician–chatbot guidance.

*H5:* Information verification utility mediates the association between perceived diagnostic support and trust in AI physician chatbot guidance.

## Materials and methods

### Study design

A cross-sectional design was employed because the study sought to characterize behavioral patterns and perceptions at a single point in time without manipulating exposure or following participants over time. This design is well suited for mapping the relationships proposed in the DHVAT framework, where the aim is empirical description rather than causal inference.

### Population

Both conditions require sustained self-management and generate ongoing information needs, making patients with diabetes or hypertension more likely to engage frequently with digital health resources.

### Sampling strategy and sample size

Participants were recruited by convenience sampling from five WhatsApp groups dedicated to diabetes and hypertension support, and one Facebook group for general chronic disease management. These communities were selected because they represent the type of digital environment in which patients with chronic diseases actively exchange health information—the same context examined in this study. The survey link was posted with a brief description of the study’s purpose, and participation was voluntary and anonymous. Two weekly follow-up reminders were sent to each patient. In total, 475 valid responses were obtained, which were sufficient for the planned descriptive and structural analyses.

### Data collection instrument

Data were collected using a purpose-built online questionnaire administered via Google Forms.

Section A collected demographic information, including age, sex, educational attainment, employment status, preferred smartphone language, daily Internet use, computer literacy, and chronic disease diagnosis. These variables were included to contextualize the participants’ digital health engagement profiles.

Section B addressed digital health information seeking and exposure. Participants indicated the frequency with which they encountered health-related content, the platforms through which they accessed it (with multiple response options to reflect real-world multi-platform behavior), and the topic categories they most commonly encountered.

Section C assessed perceptions of AI physician chatbots across four domains: diagnostic support, self-management assistance, information verification utility, and trust in AI-generated guidance. The items were rated on a five-point Likert scale (1 = strongly disagree to 5 = strongly agree). Three open-ended questions invited qualitative responses from the participants.

### Data analysis

All analyses were conducted using R. Frequencies and percentages were reported for categorical variables, and means and standard deviations were reported for Likert-scale items. Structural equation modeling (SEM) was used to examine the relationships among the four latent constructs: diagnostic support, self-management support, verification utility, and AI chatbot trust, with factor loadings and path coefficients estimated and reported. These analyses allowed examination of the relationships among the latent constructs.

The analysis was supplemented with visualization techniques to investigate the patterns of exposure to digital health information. UpSet plots are a visualization technique used to demonstrate the intersections of various digital health information sources and verification strategies used by survey participants. In contrast to traditional Venn diagrams, the UpSet visualization can display intersections among a large number of sets, making it useful for analyzing digital health engagement in multiplatform environments. This study used UpSet plots to represent the combinations of digital platforms used by participants to access health information, the methods used to verify credibility, the categories of health topics encountered in the digital environment, and the criteria used to judge the credibility of digital health information.

Qualitative responses to the open-ended questions were analyzed using thematic analysis. The initial coding of responses revealed recurring patterns. Once coded, the codes were clustered into larger conceptual groupings, from which themes and subthemes representing participants’ experiences, issues, and expectations regarding the use of AI physician chatbots emerged. Selected quotations from participants were used to highlight overarching themes and contextualize the quantitative results.

Given the cross-sectional design, all findings were descriptive and associative and no causal claims were made.

### Instrument validity and reliability

The scale items were adapted from established measures of digital health information seeking (3), technology acceptance (11, 12), and trust in AI-enabled health tools (7), with the wording modified to reflect the AI physician chatbot context. As the original instruments were developed in English and the survey was administered in Arabic, a structured cross-cultural adaptation procedure following Brislin’s translation-back-translation protocol and the WHO process for the translation and adaptation of instruments was undertaken. First, two bilingual translators (English–Arabic), one with a health informatics background and one specializing in linguistics, independently produced forward translations of all items into Modern Standard Arabic. The research team then reconciled the two forward versions into a single consensus Arabic version. Second, a different bilingual translator blinded to the original instrument performed an independent back-translation into English, which was compared with the original items to identify discrepancies in meaning. Identified inconsistencies were resolved through expert panel discussions. Third, cultural adaptation focused on adjusting examples and references to reflect the Saudi digital health ecosystem (e.g., explicit reference to the Sehhaty platform and the national 937 service) and ensuring that AI-related terminology was rendered in plain Arabic comprehensible to non-specialist patients. Fourth, the adapted Arabic version was reviewed by a panel of three experts (two health informatics academics and one practizing primary-care physician) for content validity, and a content validity index (I-CVI) of ≥ 0.80 was achieved for all retained items. Content validity was established through a review by two health informatics researchers prior to pilot testing with 15 individuals from the target population; minor wording adjustments were made based on their feedback. Internal consistency was assessed using Cronbach’s alpha.

Cronbach’s alpha indicated high internal consistency for the 14-item AI Chatbot Perceptions Scale (see Table 1), the scale could be considered to be very highly reliable (α = 0.90). The corrected item-total correlation values ranged from 0.40 to 0.73, and “alpha if item deleted” values ranged from 0.89 to 0.90, indicating that none of the individual survey items would contribute significantly to enhancing the overall reliability of the scale if deleted. These findings indicate that the items consistently measured different aspects of AI chatbot diagnostic support, self-management assistance, information validation, and user trust.

**TABLE 1:** Self-diagnosis, self-management, verification, and trust in an AI physician chatbot.

Item	Mean	SD	Strongly disagree	Disagree	Neutral	Agree	Strongly agree
The AI physician chatbot helps me understand whether my symptoms may require medical attention.	3.66	1.12	29 (6.1%)	46 (9.7%)	93 (19.7%)	193 (40.8%)	112 (23.7%)
Using the AI physician chatbot improves my ability to recognize early signs related to my chronic condition.	3.67	1.07	19 (4.1%)	40 (8.5%)	135 (28.8%)	158 (33.8%)	116 (24.8%)
The AI physician chatbot provides clear guidance that supports initial self-diagnosis decisions.	3.79	1.07	18 (3.8%)	34 (7.2%)	119 (25.1%)	161 (33.9%)	143 (30.1%)
I feel more confident assessing my symptoms after using the AI physician chatbot.	3.26	1.31	48 (10.1%)	97 (20.5%)	133 (28.1%)	77 (16.2%)	119 (25.1%)
The AI physician chatbot helps me manage my chronic condition more effectively on a daily basis.	3.54	1.17	35 (7.4%)	51 (10.8%)	118 (25.0%)	162 (34.3%)	106 (22.5%)
Using the AI physician chatbot supports my adherence to treatment or lifestyle recommendations.	3.45	1.27	53 (11.2%)	44 (9.3%)	132 (28.0%)	124 (26.3%)	119 (25.2%)
The AI physician chatbot provides useful advice for monitoring my chronic condition outside clinic visits.	3.27	1.39	75 (15.8%)	61 (12.8%)	120 (25.3%)	97 (20.4%)	122 (25.7%)
The AI physician chatbot helps me decide when self-care is sufficient and when professional care is needed.	3.1	1.39	95 (20.0%)	56 (11.8%)	128 (26.9%)	100 (21.1%)	96 (20.2%)
I use the AI physician chatbot to verify health information I encounter on social media or online platforms.	3.48	1.28	57 (12.0%)	40 (8.4%)	118 (24.8%)	139 (29.3%)	121 (25.5%)
The AI physician chatbot helps me distinguish between reliable health information and misinformation.	3.25	1.38	75 (15.8%)	65 (13.7%)	115 (24.2%)	105 (22.1%)	115 (24.2%)
Using the AI physician chatbot reduces my reliance on unverified health advice from informal sources.	3.74	1.13	26 (5.6%)	29 (6.2%)	127 (27.3%)	139 (29.9%)	144 (31.0%)
I trust the medical information provided by the AI physician chatbot.	3.29	1.3	61 (13.0%)	58 (12.3%)	142 (30.1%)	103 (21.9%)	107 (22.7%)
I consider the AI physician chatbot to be a reliable source of health information.	3.44	1.38	69 (14.7%)	41 (8.7%)	114 (24.3%)	105 (22.4%)	140 (29.9%)
I feel comfortable relying on the AI physician chatbot for health-related guidance alongside professional care.	3.86	1.11	22 (4.6%)	31 (6.5%)	104 (21.9%)	152 (32.0%)	166 (34.9%)

### Ethics statement

The study was granted ethical approval from Qassim University’s Research Ethics Committee (Approval no. 23-19-01) prior to conducting the research. All ethical principles outlined in the Declaration of Helsinki and the Institutional Guidelines for Research Involving Human Subjects were respected during this study. Participation was voluntary, and participants received information about the nature and purpose of the study, the type of questions to be asked, and their right to withdraw at any time without consequences. All participants provided electronic consent prior to completing the questionnaire; no personally identifiable information was captured, and responses were collected via an anonymous online survey platform. The data were securely stored and available only for research purposes.

## Results

### Demographic and digital engagement characteristics

**FIGURE 2:**
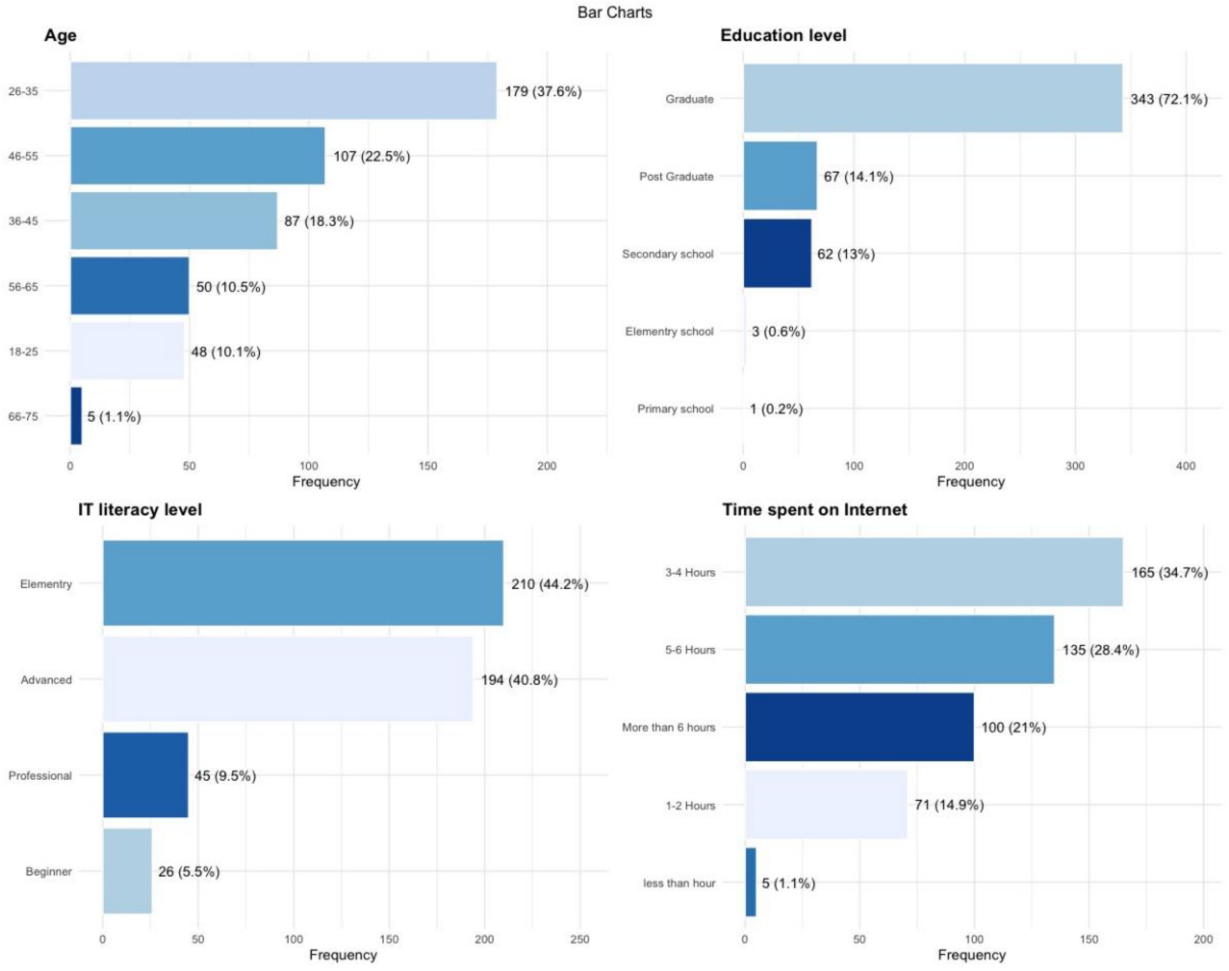
Demographic characteristics of the study participants.

Figure 2 shows the participants’ demographic and digital engagement characteristics under multiple response categories. The largest age segment was between the ages of 26–35 (*n* = 179, 37.6%), followed by those aged 46–55 (*n* = 107, 22.5%), and those aged 36–45 (*n* = 87, 18.3%). A smaller percentage of participants fell within the age groups of 56–65 years (*n* = 50, 10.5%), 18–25 (*n* = 48, 10.1%), and 66–75 (*n* = 5, 1.1%). Most participants had graduate-level education (*n* = 343, 72.1%), followed by participants with postgraduate qualifications (*n* = 67, 14.1%) and those who had only completed secondary education (*n* = 62, 13.0%). Only 7 participants (1.5%) had completed elementary or primary education. Digital skills were reported to be at the elementary IT literacy level for the largest number of participants (*n* = 210, 44.2%), with an almost equal number of participants reporting their level of IT literacy to be at the advanced level (*n* = 194, 40.8%), followed by the professional level (*n* = 45, 9.5%) and beginner level (*n* = 26, 5.5%). A high level of daily Internet usage was reported by participants; those who used the internet for 3–4 h per day constituted the largest percentage of respondents (*n* = 165, 34.7%), while the next largest percentage of participants used the Internet for 5–6 h per day (*n* = 135, 28.4%). Overall, 100 participants (21.0%) reported using the internet for more than 6 h per day, 71 (14.9%) reported using it for 1–2 h per day, and 5 (1.1%) reported using it for < 1 h per day.

**FIGURE 3:**
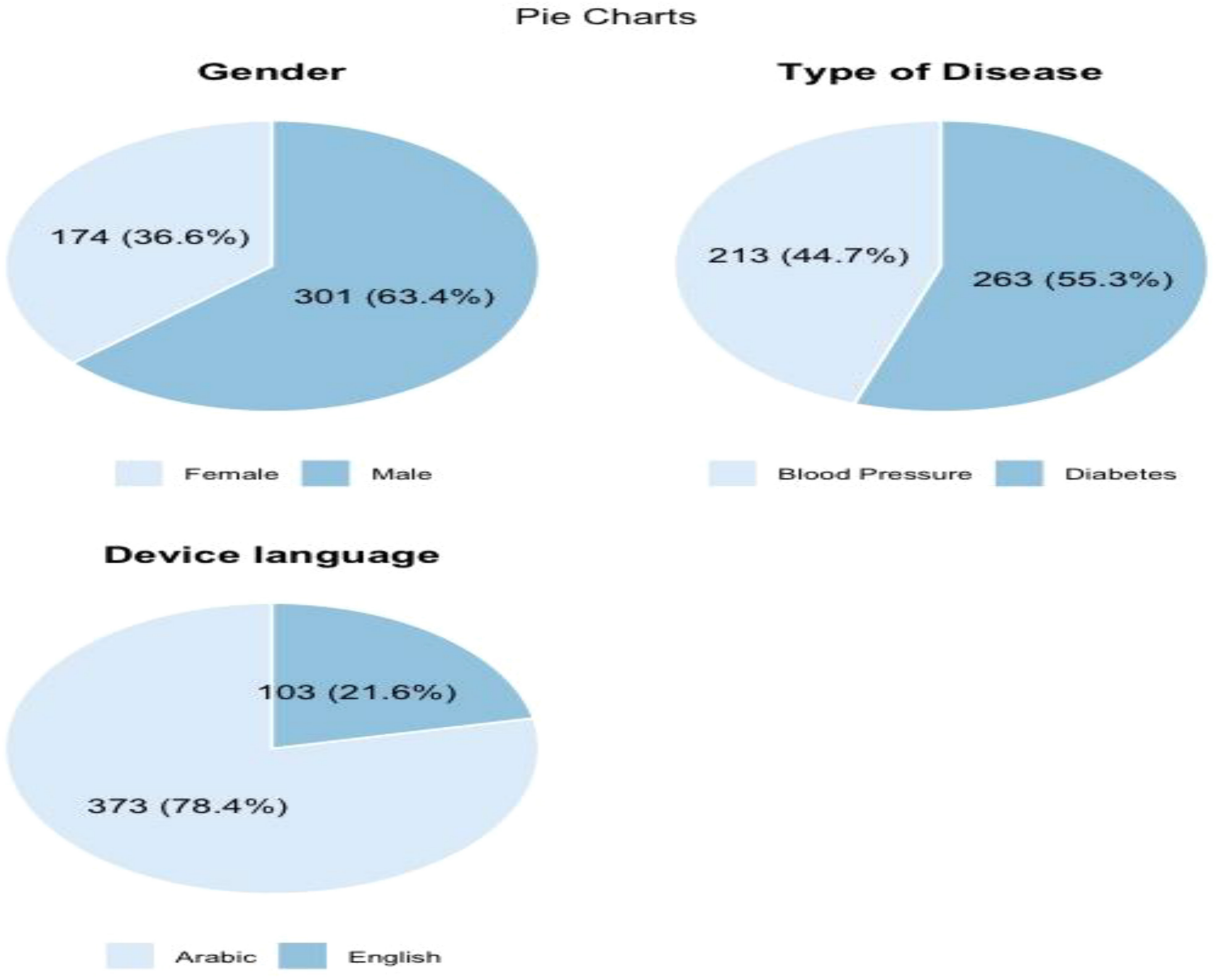
Gender, type of disease and smartphone language preferences among study participants.

The participants’ demographics are presented in Figure 3, which illustrates the four main binary demographic variables examined in this study. The majority of the respondents were male (*n* = 301, 63.4%), and almost one-third were female (*n* = 174, 36.6%). The proportion of participants who reported having diabetes (*n* = 263, 55.3%) was higher than the proportion who reported having hypertension (*n* = 213, 44.7%). Most respondents reported having smartphone interactions in Arabic (*n* = 373, 78.4%), with only a small minority (*n* = 103, 21.6%) interacting in English. This indicates that most participants in this study were male, had diabetes rather than hypertension, and used Arabic as the most common language for their smartphone interactions.

### Health information use and clarification behaviors

**FIGURE 4:**
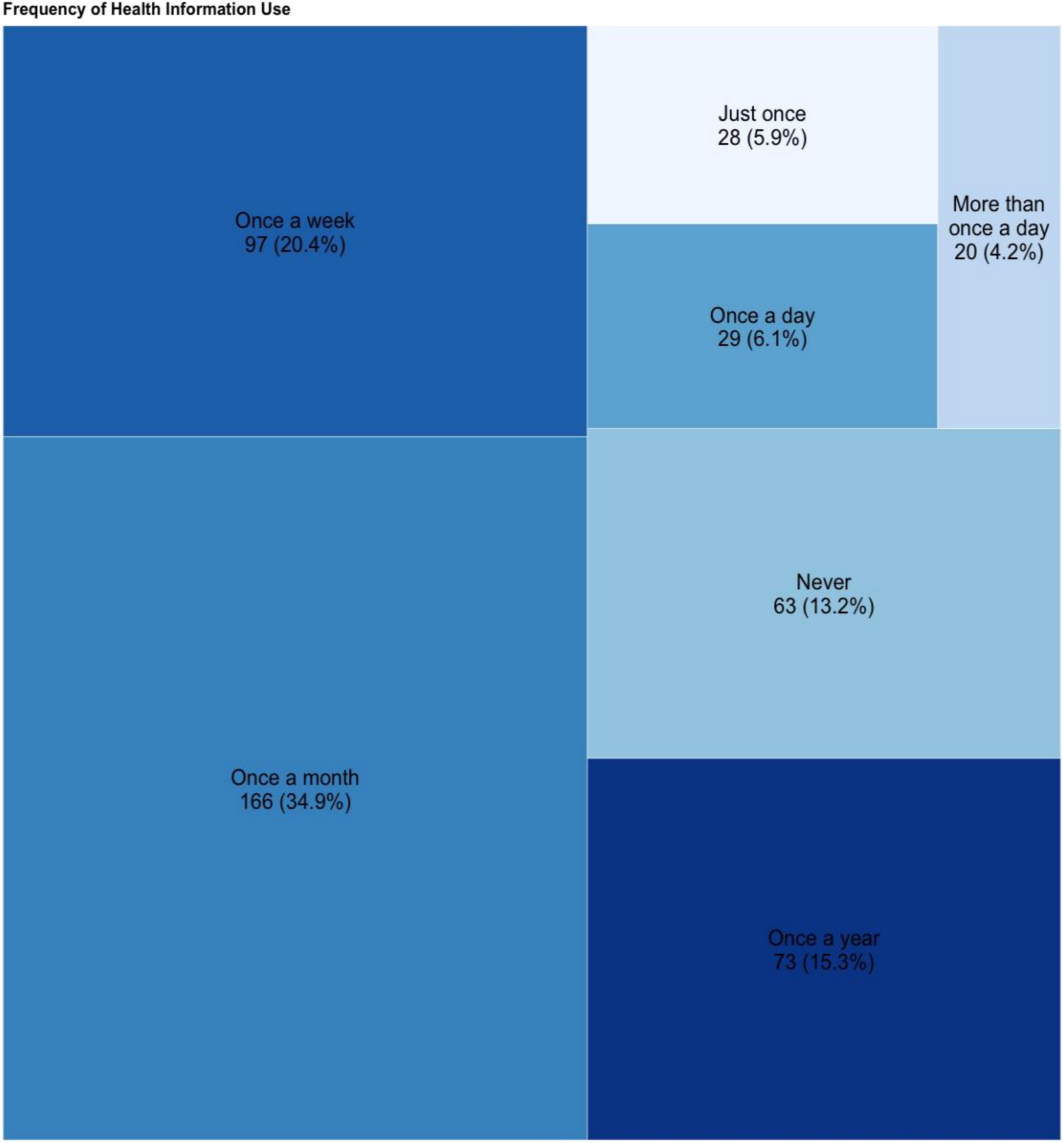
Frequency of health information messages.

As shown in Figure 4, the frequency of health information use varied across the participants. As can be seen from the results, the most common pattern was occasional use, with once-a-month engagement reported by 166 participants (34.9%), followed by once-a-week use (97 participants, 20.4%). Regular use was less common, with only 29 participants (6.1%) reporting daily use and 20 (4.2%) reporting use more than once a day. However, a notable proportion of participants reported limited engagement, including those who used health information only once (28 participants, 5.9%) or never (63 participants, 13.2%).

**FIGURE 5:**
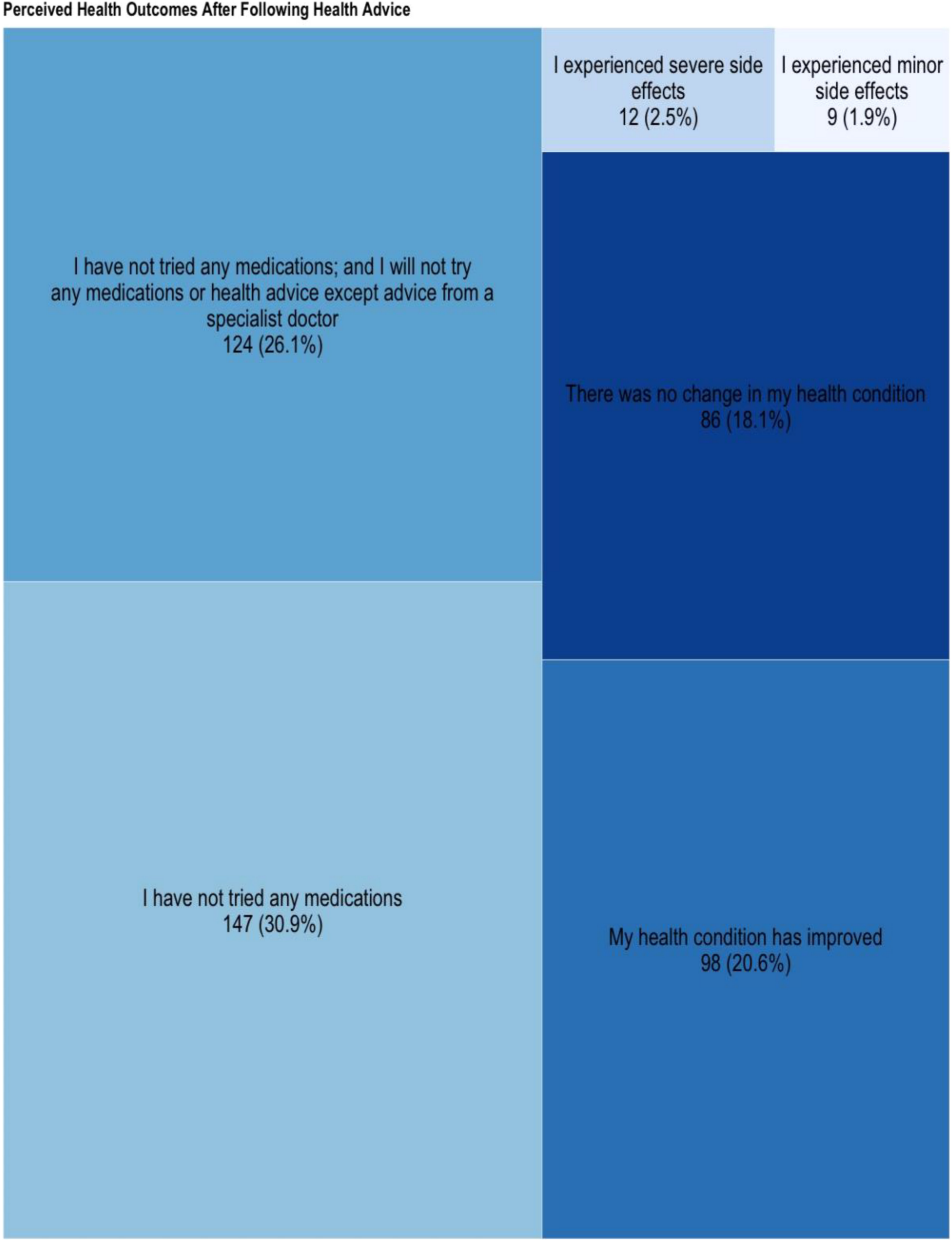
Perceived health outcomes after following health advice.

Figure 5 summarizes the perceived health outcomes after following health advice. In this regard, the largest group reported not having tried medications and relying instead on specialist advice (124 participants, 26.1%), while 147 participants (30.9%) reported not trying any medications. In contrast, 98 participants (20.6%) reported an improvement in their health condition, whereas 86 (18.1%) reported no change. Importantly, reports of adverse outcomes were relatively uncommon, with minor side effects reported by 9 participants (1.9%) and severe side effects reported by 12 participants (2.5%).

**FIGURE 6:**
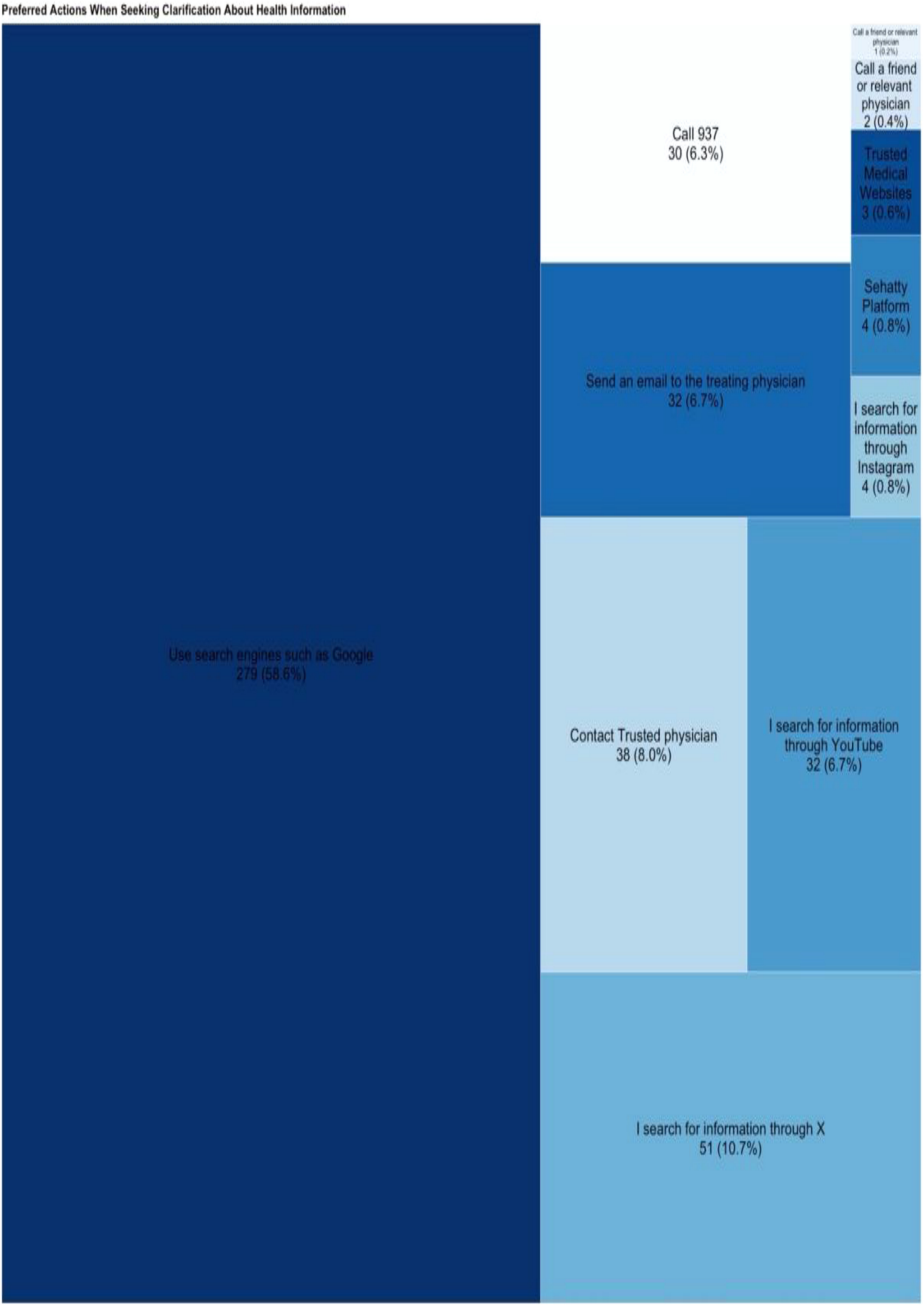
Preferred actions when seeking clarification about health information.

As illustrated in Figure 6, the participants’ preferred actions when seeking clarification about health information showed a clear pattern. In particular, the majority relied on general search engines, such as Google (279 participants, 58.5%). A smaller proportion reported consulting trusted physicians (38 participants, 8.0%) or contacting the national 937 service (30 participants, 6.3%). However, the use of official digital platforms, including Sehhaty and trusted medical websites, was limited. Taken together, these findings suggest that, while participants actively seek health information, reliance on informal digital sources remains dominant, highlighting the potential value of trusted AI-enabled tools for guidance and verification.

### AI chatbot perceptions and structural equation modeling

Table 1 reports participants’ responses to the Likert-scale items. As can be seen from the table, mean scores for self-diagnosis items ranged from 3.26 to 3.79. The highest mean was observed for receiving clear guidance to support initial self-diagnosis decisions (mean = 3.79), with 64.0% of participants selecting agree or strongly agree. However, confidence in assessing symptoms showed lower agreement, with only 41.3% reporting agreement and a substantial neutral response (28.1%). With regard to self-management, mean scores ranged from 3.10 to 3.54. Daily management support received higher agreement (56.8%) than decision-making about when professional care was required (41.3%). Turning to verification of health information, reducing reliance on unverified sources showed one of the strongest responses (mean = 3.74), with 60.9% agreeing or strongly agreeing. Moreover, trust-related items showed mixed responses. Trust in the chatbot alone had a mean of 3.29, whereas comfort using it alongside professional care was higher (mean = 3.86), with 66.9% of participants expressing agreement. Taken together, these results show stronger endorsement for supportive and complementary uses of the AI physician chatbot than for independent decision-making.

**FIGURE 7:**
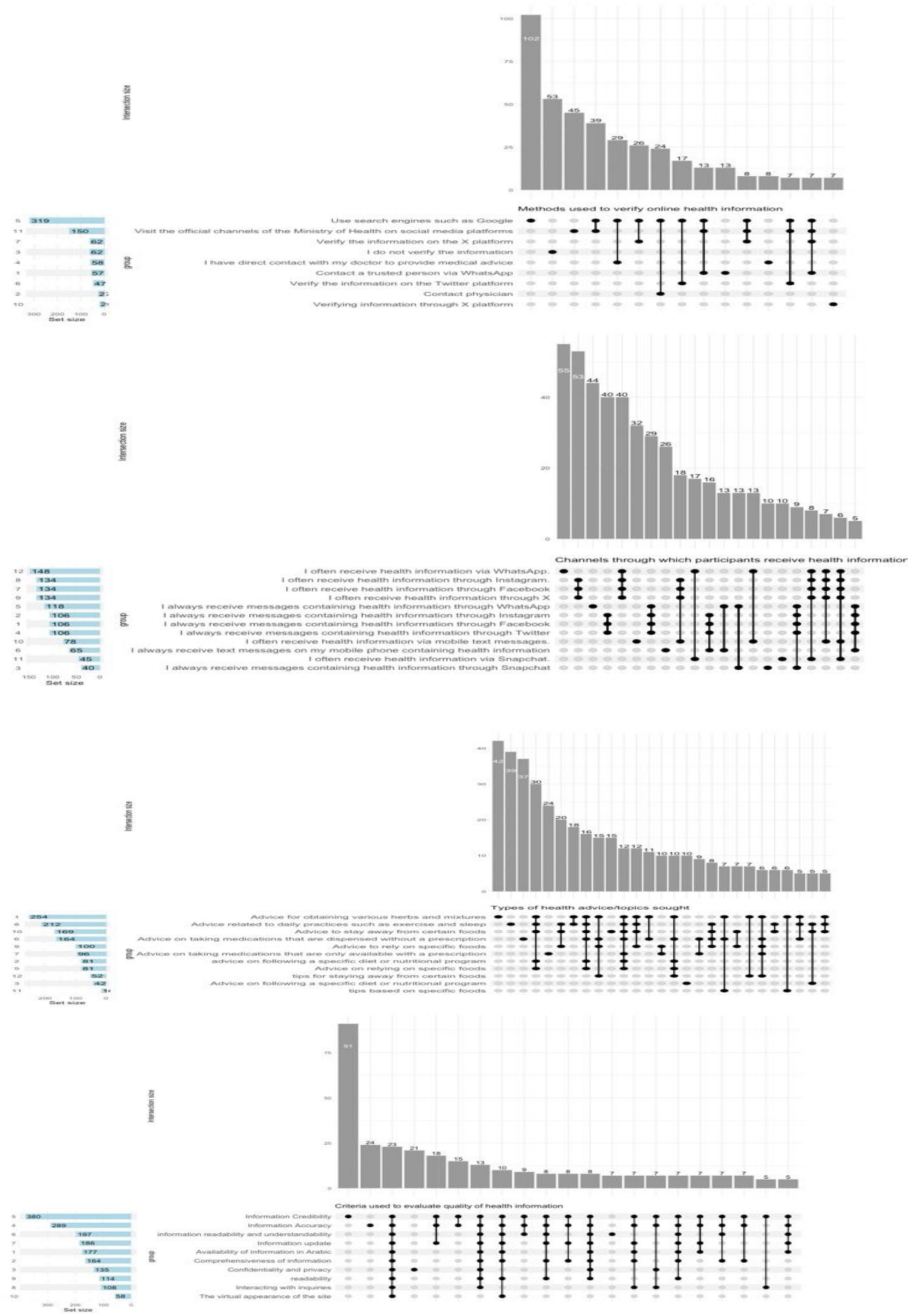
UpSet plots showing the frequency and intersection patterns of participants’ digital health information sources, verification behaviors, perceived benefits, and evaluation criteria when interacting with online health information and AI-enabled digital.

Taken together, the four UpSet plots shown in Figure 7 indicate clear patterns in how participants sought, verified, and evaluated online health information. In the upset plot of information channels, WhatsApp clearly dominated, with the largest intersection (≈48–50%) indicating frequent receipt of health information through this platform, either alone or in combination with others such as YouTube and X; moreover, multi-platform exposure was common, suggesting layered information environments rather than single-source reliance. Turning to verification practices, the upset plot shows that searching via Google and visiting official channels were the most frequent strategies (approximately 50 and 27%, respectively), while direct verification through physicians or trusted individuals appeared less often. However, notable intersections revealed that participants often combined formal and informal checks rather than relying on one method. With respect to topics sought, the upset plot highlights a strong emphasis on lifestyle-related advice, particularly diet, exercise, sleep, and herbal remedies, which together formed the largest intersections (≈30–35%); by contrast, prescription-related queries appeared less frequently and mainly in combination with other topics. Finally, the upset plot on evaluation criteria demonstrates that credibility-based factors were prioritized, with information credibility, source accuracy, and clarity consistently appearing in the largest intersections (approximately 35–40%); nevertheless, visual appearance and popularity still co-occurred with credibility factors, indicating that surface cues were not ignored. Overall, these upset plots suggest that participants engage in selective yet multichannel health information seeking, combine verification strategies, and apply layered criteria when judging trustworthiness.

**FIGURE 8:**
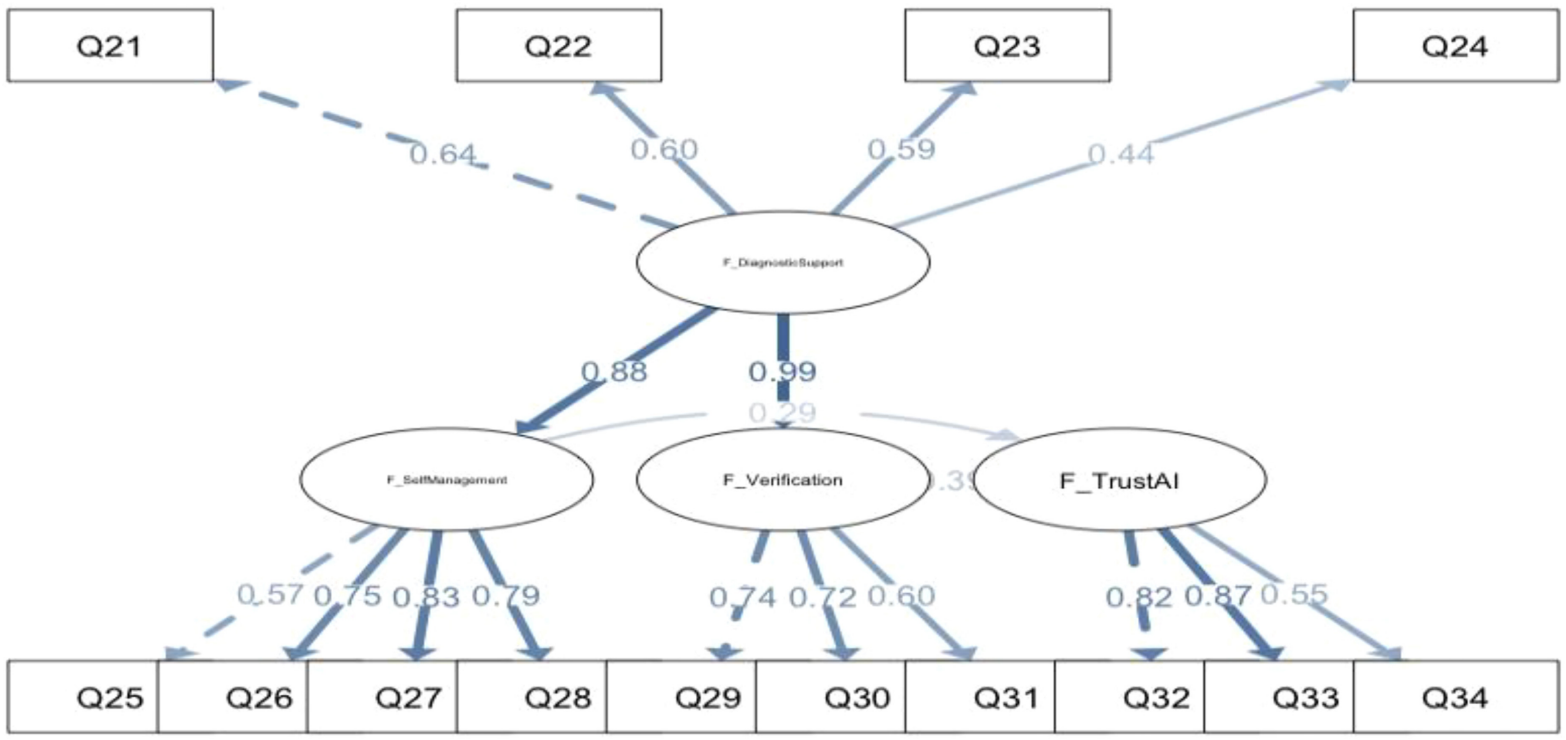
Structural equation model illustrating the relationships between diagnostic support, self-management support, information verification utility, and trust in AI physician chatbots. Standardized factor loadings and path coefficients are displayed.

Figure 8 shows the relationships between the four latent constructs derived from the AI physician chatbot perception scale, which were studied using a structural equation model: diagnostic support, self-management support, information verification utility, and trust in AI chatbot guidance. The measurement model demonstrated adequate loadings of the indicators on the constructs. The factor loadings for diagnostic support ranged from 0.44 to 0.64. Self-management support had higher factor loadings between 0.50 and 0.83. The verification utility construct had factor loadings between 0.60 and 0.74, and trust in AI had factor loadings between 0.55 and 0.87. This demonstrates that the items accurately measured AI chatbot support and trust from participants’ perspectives.

Although one indicator of the diagnostic support construct loaded at 0.44, slightly below the conventional 0.50 threshold often cited for confirmatory factor analysis, this item was retained in the final measurement model for three substantive and methodological reasons. First, the item was theoretically central to the diagnostic support construct as defined by the DHVAT framework; it captured patients’ confidence in independently assessing their own symptoms after using an AI chatbot, which is a core behavioral manifestation of diagnostic support that cannot be adequately represented by the remaining items without losing construct breadth. Second, contemporary psychometric guidance in social and health sciences (e.g., Hair et al., 2019; Kline, 2023) recognizes that loadings between 0.40 and 0.50 may be retained when the indicator contributes meaningfully to content coverage and when overall construct reliability and convergent validity remain acceptable; in the present model, composite reliability for the diagnostic support construct exceeded 0.70 and average variance extracted (AVE) approached the conventional cut-off of 0.50, supporting retention. Third, a sensitivity analysis was conducted in which the model was re-estimated with this indicator removed; path coefficients between latent constructs changed by < 0.03 in absolute terms, indicating that the substantive findings reported here are robust to this specification choice. Therefore, the decision to retain this item reflected a balance between strict statistical thresholds and the need to preserve the theoretical meaningfulness of the construct.

The central position of diagnostic support in the pattern of associations among the participants’ perceptions of AI chatbot usefulness was reflected in the structural model. The pathway from diagnostic to self-management support and verification utility was strongly positive. Self-management was positively associated with trust in the AI chatbot guidance, with a standardized coefficient of 0.29, and verification utility showed a standardized coefficient of 0.39 with trust. Within the cross-sectional design adopted here, these coefficients describe associative rather than causal relationships: participants who reported that the chatbot supported symptom assessment were more likely to report using it for self-management and verification, and these perceptions were, in turn, associated with higher levels of expressed trust in chatbot-provided guidance. No temporal ordering or causal direction was inferred from the data.

### Responses to open ended questions

Analysis of the open-ended responses provided a clearer understanding of how participants viewed AI physician chatbots as a tool to support their decision-making process regarding health, as well as their reactions to chatbot technology. Many participants noted that these chatbots were beneficial in assisting them in making sense of symptoms and gaining an understanding of what types of health issues could exist before seeking professional help (*n* = 22). As one participant stated, “The chatbot helps me to better understand what my symptoms could possibly be.” Participants indicated that the chatbot’s guidance helped them decide whether to seek medical treatment and assess the urgency of their condition (*n* = 19). Another benefit expressed by several participants was the convenience and accessibility of chatbots, as many chatbots allow patients to find immediate health information instead of having to schedule a clinical appointment (*n* = 21). A number of respondents also noted that the conversational interface makes it easier for patients to ask follow-up questions (*n* = 16) and that many chatbots provide information in lay terms (*n* = 20). However, the majority of respondents clearly stated that chatbots should never be used as a replacement for professional diagnoses (*n* = 23), suggesting that most respondents viewed chatbots as supplemental resources rather than replacements for traditional healthcare (see Supplementary Appendix Table 1).

Trust emerged as an important factor in determining how participants perceived AI-generated health information, with multiple factors contributing to the development and enhancement of trust. Specifically, trust was strengthened when the advice given by the chatbots corresponded to suggestions made by medical professionals (*n* = 25) and was supported by evidence-based research (*n* = 23). Additionally, many respondents stressed the importance of transparency in terms of sources of information (*n* = 18) and expressed concerns over protecting personal data and private information (*n* = 24). Respondents also voiced concerns regarding the possibility of receiving misleading advice from chatbots (*n* = 22), as well as the danger of becoming reliant on automated systems (*n* = 15); for instance, one respondent cautioned that “misleading advice can cause significant harm.” In addition, most respondents viewed chatbots as tools that work alongside healthcare professionals rather than as replacements for consultation and evaluation by a healthcare provider (*n* = 24; *n* = 22). Additionally, participants recommended stricter institutional oversight, ethical governance, and continued evaluation of AI technologies in healthcare to support responsible.

## Discussion

The findings reveal that adults managing diabetes or hypertension in Saudi Arabia engage with digital health information through multiple overlapping channels, approach that information with a degree of skepticism, and treat AI-enabled tools as a means of verification rather than as a replacement for clinical advice. Integrating the survey data with open-ended responses provides a more complete picture of how patients navigate today.

The participants reported using AI-enabled chatbot systems to confirm and verify their health information. Through the use of AI physician chatbots, participants reported that chatbots assisted in determining which symptoms might be indicative of the need for medical attention and guided users in making early health-related decisions. However, the participants generally indicated that their degree of confidence in their own ability to assess their symptoms independently was lower than their confidence in the professional judgment of a physician in determining the type of medical care needed. The current findings and previous studies on interactive voice response (IVR) systems and chatbot technology demonstrate similar patterns in the use of these systems for early symptom assessment, triage, and decision-making support prior to seeking medical consultation (6, 14).

The findings from this study illustrate a potential connection between people’s trust in AI-generated health advice and their behaviors when evaluating the reliability of information gathered through online health resources and whether to act on it. The participants used various methods to evaluate the credibility of health-related online sources, including search engines, government-sponsored health-related websites, and discussions with healthcare providers on specific symptoms, medications, and treatment options. AI-enabled physician chatbots might provide an additional tool for individuals to use when verifying the integrity of health-related information collected from online sources, and could help reduce users’ reliance on unverified information gathered from social media and other digital sources. Additionally, the behaviors of the participants in this study demonstrated a multistep verification process while evaluating their online health information and purchasing medications using digital health applications, demonstrating the importance of digital health literacy (5). Nevertheless, trust in AI-generated health advice remains at a moderate level, indicating the need to address transparency, reliability, and the appropriate use of algorithm-driven health systems (7). The participants’ preference for receiving chatbot guidance in combination with healthcare provider care indicates the importance of integrating chatbot technology within trusted healthcare systems and platforms.

The cultural context of Saudi Arabia provides a unique perspective for the evaluation and potential integration of AI-related digital health technologies. Nationally sponsored digital health services may expand rapidly but still show fluctuating levels of sustained participation (8). Within the present analysis, the concurrent use of both AI physician chatbots and national mobile health applications served as part of the information verification strategy for participants. In fact, the use of AI-enabled physician chatbots to help alleviate uncertainty and provide assistance with symptom identification is well supported by the qualitative evidence generated by this analysis. However, this support is tempered by the need for professional oversight, institutional endorsement, and transparent governance mechanisms for digital health applications and technology.

Overall, AI-enabled physician chatbots function as supporting elements within a digital health ecosystem that enable patients to effectively interpret their symptoms and verify the validity of the information gathered through online resources. However, patients with chronic illnesses still rely heavily on healthcare providers when deciding on an appropriate course of action. Integrating AI-guided decision-support systems into trusted systems developed by healthcare organizations may contribute to enhanced reliability and accuracy in the verification of digital health information, improve patient awareness of chronic disease management strategies, preserve transparency and public confidence in the healthcare system, and improve the reliability of information provided to patients.

These findings have direct implications for managing health misinformation in populations with chronic diseases. The dominance of WhatsApp and social media as primary channels through which participants encountered health content, combined with their relatively limited use of official platforms such as Sehhaty for clarification, indicates that misinformation can circulate widely before reaching any verified counter message. Within the DHVAT framework, this is precisely the point at which verification behavior becomes clinically consequential: when patients with diabetes or hypertension act on inaccurate information about diet, herbal remedies, or medication interactions, the downstream risks include glycemic instability, hypertensive episodes, and inappropriate self-medication. The observation that participants used AI physician chatbots largely to corroborate or challenge information encountered elsewhere suggests that generative AI tools could play a meaningful role in countering misinformation but only if their outputs are grounded in evidence-based sources and accompanied by transparent uncertainty disclosures. Without such safeguards, AI chatbots risk amplifying the misinformation they intend to address, particularly because their fluent and confident responses can give inaccurate content an unwarranted appearance of authority. Therefore, public health strategies should treat AI chatbots as one component of a broader misinformation resilience system that includes clinician communication, official platform investment, and digital health literacy education.

The second set of implications concerns regulatory and governance gaps surrounding the use of AI chatbots in healthcare. Unlike traditional medical devices and clinical decision-support software, generative AI assistants used for health-related queries currently occupy an uncertain regulatory space in most jurisdictions, including Saudi Arabia. Participants in this study repeatedly emphasized the need for institutional endorsement, professional oversight, and clear boundaries on the role of AI tools-preferences that align with emerging regulatory developments such as the European Union AI Act, the United States Food and Drug Administration’s evolving guidance on adaptive AI/ML-based software as a medical device, and the World Health Organization’s recent recommendations on the ethics and governance of large multimodal models in health. National digital health strategies in Saudi Arabia, including those administered by the Saudi Data and Artificial Intelligence Authority (SDAIA) and the Ministry of Health, will need to articulate clearer rules regarding the scope of permitted clinical claims, mandatory disclaimers, audit and accountability mechanisms, and integration pathways with regulated platforms such as Sehhaty. The findings of the present study suggest that patients themselves are receptive to, and indeed actively request, this kind of governance infrastructure, which represents a meaningful policy opportunity rather than a barrier to adoption.

From an ethical perspective, the moderate trust scores reported in this study should be interpreted as an opportunity rather than a deficiency. Calibrated trust, in which patients place confidence in AI tools commensurate with their actual reliability for a given task, is preferable to either reflexive rejection or uncritical reliance. The qualitative responses suggested that the participants were attentive to several genuine ethical concerns, including data privacy, transparency about information sources, the risk of misleading advice, and the danger of overreliance on automated systems. These concerns map directly onto core principles of AI ethics frameworks: autonomy (patients retaining decision-making authority), non-maleficence (avoiding harm from inaccurate advice), justice (equitable access and outcomes), explicability (understandable reasoning), and privacy (protection of sensitive health data). Particularly relevant for populations with chronic diseases is the risk that habitual reliance on AI chatbots for symptom interpretation could erode patients’ help-seeking thresholds, delaying clinical consultation in situations where timely professional assessment is critical (e.g., evolving cardiovascular symptoms in hypertensive patients or ketoacidosis warning signs in patients with diabetes). System-level safeguards, including explicit referral prompts and integration with national triage services, are therefore essential to ensure that AI-enabled chatbots support rather than substitute for clinical judgment.

Finally, the public health relevance of these findings cannot be separated from the issues of health equity and digital access. The verification and trust patterns described here were generated by a sample with relatively high digital engagement, and they may not be generalizable to chronic disease patients with limited literacy, restricted internet access, low-end devices, or limited Arabic language support for advanced AI tools. Older adults in rural regions of the Kingdom, individuals with lower educational attainment, and patients with sensory or cognitive impairments may face structural barriers to using AI chatbots safely and effectively, even when the technology itself is freely available. If AI-enabled mHealth tools are integrated into mainstream chronic disease pathways without parallel investment in digital inclusion, there is a meaningful risk that they will widen, rather than narrow, existing health disparities. Equitable deployment requires attention to language and dialect coverage, voice-based and low-bandwidth interfaces, accessibility features, and digital health literacy programs targeted at populations currently underserved by digital health ecosystems.

From a theoretical perspective, this study extends existing models of digital health technology adoption by introducing verification behavior as an intermediary mechanism between digital health information exposure and trust in AI-enabled decision-support tools. By integrating Wilson’s Information Behavior Model with TAM and UTAUT2, the proposed DHVAT framework offers a structured explanation of how patients evaluate and incorporate AI-generated health guidance within broader digital health ecosystems.

### Study strengths and limitations

A key strength of this study is the integration of quantitative survey data with qualitative responses, allowing a more comprehensive understanding of both the prevalence and underlying meanings of digital health behaviors. Nevertheless, this study has several limitations. First, the cross-sectional design limits the ability to establish causal relationships between digital health information exposure, AI tool use, and health-related decision making. Accordingly, the structural relationships reported between diagnostic support, self-management support, verification utility, and trust should be interpreted as patterns of association, rather than as evidence of a causal direction. Second, the sampling strategy is subject to important constraints regarding its generalizability. Participants were recruited via convenience sampling from chronic disease support communities on WhatsApp and Facebook, which inevitably over-represented individuals who were already digitally literate, regularly online, and engaged in peer-to-peer health information exchange. Patients with chronic diseases who do not use social messaging platforms, have limited smartphone access, rely on family members for digital interactions, or reside in areas with poor connectivity are systematically under-represented. Therefore, the verification and trust behaviors documented here characterize a digitally engaged segment of the chronic disease population in Saudi Arabia, and should not be extrapolated to less tech-savvy patients without further targeted research. Third, the male majority in the achieved sample may reflect both gender differences in social media participation patterns and the recruitment channels used and may limit the applicability of the findings to female chronic-disease populations who carry a substantial share of household health-information work. Fourth, self-reported behavioral measures are vulnerable to social desirability bias, particularly with respect to verification activities. Participants may overstate the extent to which they cross-check online health information against authoritative sources. Fifth, although the AI chatbot construct was operationalized in this study by referring to generative AI conversational agents (rather than rule-based symptom checkers), participants in everyday practice may interact with both categories of tools, and their reported perceptions may incorporate experiences with hybrid systems that this study cannot fully disentangle. Finally, the study was conducted within a single national context with its own digital health ecosystem and regulatory environment; cross-cultural and cross-system replication is needed to assess the boundary conditions of the DHVAT framework.

### Recommendation for future researchers

Future research should explore pathways for incorporating AI-based digital health technology into routine healthcare delivery. Research on AI-based digital health technology should also be conducted in conjunction with the measurement of outcomes and evaluation of how long users will continue to use AI-supported health and wellbeing information technologies. Comparative research across healthcare systems and cultural settings may also inform best-practice protocols for the safe and effective dissemination of AI-enhanced diagnostic decision-support technology.

## Conclusion

In conclusion, this study demonstrates that digital health information-seeking among individuals with chronic diseases is characterized by fragmented exposure across multiple online platforms and cautious engagement with AI-enabled health tools. Many participants reported using AI physician chatbots and national mHealth services primarily to verify and clarify health information encountered online rather than as substitutes for professional medical care. These findings highlight the importance of verification behavior as a key mechanism in shaping digital health decision-making. The results further suggest that AI-enabled mHealth tools may function most effectively as supportive decision-support systems integrated within existing healthcare services. From a digital public health perspective, designing systems that facilitate information verification, transparency, and clinician-supported guidance may strengthen patient trust while supporting informed health-related decisions.

## Data Availability

The raw data supporting the conclusions of this article will be made available by the authors, without undue reservation.
