# Supplementary material for: Trust and verification in AI-enabled physician chatbots for chronic disease management: evidence from digital health behavior
Source: Front Med (Lausanne). 2026 Jun 24;13:1830356. doi: 10.3389/fmed.2026.1830356 (PMC13341845; doi:10.3389/fmed.2026.1830356)
Supplement: Supplementary file 1 [file Supplementary_file_1.docx]

Table 1: Themes, sub-themes, frequency of responses, and illustrative quotes from open-ended questions on AI-based doctor chatbots

| Theme | Sub-theme | No. of responses (n) | Illustrative quotes |
| --- | --- | --- | --- |
| Q1. Experience and perceptions | | | |
| Perceived usefulness for initial health understanding | Clarifying symptoms and possible causes | 22 | “It helps me understand what my symptoms might be related to.”; “The chatbot gives me an idea of what could be causing my symptoms.”; “It makes symptoms easier to interpret before I panic.” |
|  | Supporting decisions about seeking care | 19 | “It helps me decide whether I really need to see a doctor.”; “After using it, I can judge if the issue is urgent or not.”; “It supports my decision on whether to seek medical advice.” |
|  | Acting as a first point of reference | 17 | “I usually use it as my first step before anything else.”; “It works as an initial source of health information.”; “It is often the first place I go when I have a question.” |
|  | Reducing uncertainty around minor issues | 14 | “It reassures me when the problem seems minor.”; “It helps reduce unnecessary worry.”; “For small issues, it gives enough information to feel comfortable.” |
| Ease of use and accessibility | Immediate access and availability | 21 | “I can use it anytime without waiting.”; “It is available whenever I need quick information.”; “There is no need for appointments or delays.” |
|  | User-friendly interaction | 18 | “The interaction feels simple and straightforward.”; “It is easy to ask questions in normal language.”; “I find it more user-friendly than medical websites.” |
|  | Ability to ask follow-up questions | 16 | “I like that I can ask follow-up questions immediately.”; “It allows me to clarify things I do not understand.”; “The conversation style makes it easier to explore topics.” |
|  | Convenience compared with traditional sources | 13 | “It is more convenient than searching multiple websites.”; “Everything is in one place, which saves time.”; “It feels faster than reading long medical articles.” |
| Clarity and understandability of information | Use of simple, non-technical language | 20 | “The language is easy to understand.”; “It explains things without complicated medical terms.”; “I appreciate how simple the explanations are.” |
|  | Step-by-step explanations | 15 | “It explains information step by step.”; “The structured explanations make things clearer.”; “It breaks complex information into smaller parts.” |
|  | Clear summaries of health information | 12 | “The summaries are clear and helpful.”; “It gives a good overview without too much detail.”; “The information is concise and to the point.” |
| Emotional reassurance and confidence-building | Reduction of anxiety | 14 | “It reduces my anxiety when I am worried.”; “Sometimes it helps calm me down.”; “It reassures me while I wait to see a doctor.” |
|  | Feeling supported | 11 | “It feels like having someone guide me.”; “It provides a sense of support when I have concerns.”; “It makes me feel less alone with health questions.” |
|  | Increased confidence in asking questions | 9 | “I feel more confident asking questions to AI.”; “It helps me ask things I hesitate to ask doctors.”; “I feel less judged when using it.” |
| Awareness of limitations | Lack of personalisation | 16 | “The advice sometimes feels too general.”; “It does not always consider personal differences.”; “It cannot fully personalise responses.” |
|  | Inability to replace professional diagnosis | 23 | “It cannot replace a real doctor.”; “I would never rely on it for diagnosis.”; “Professional medical advice is still necessary.” |
|  | Risk of oversimplification | 10 | “Some explanations feel oversimplified.”; “Complex issues are not always well covered.”; “It works better for simple questions.” |
| Q2. Trust and concerns | | | |
| Accuracy and reliability of information | Consistency with professional advice | 25 | “I trust it more when it matches what doctors say.”; “Consistency with medical advice increases my trust.”; “If it agrees with professional guidance, I feel reassured.” |
|  | Evidence-based recommendations | 23 | “It should be based on medical evidence.”; “Evidence-based advice makes it trustworthy.”; “Medical guidelines increase my confidence.” |
|  | Avoidance of contradictory information | 17 | “Contradictory advice reduces my trust.”; “Conflicting information makes me unsure.”; “Consistency is essential for trust.” |
| Transparency of sources and limitations | Clear disclosure of sources | 18 | “It should clearly state where information comes from.”; “Referencing sources increases my confidence.”; “I trust it more when sources are mentioned.” |
|  | Acknowledgement of uncertainty | 14 | “I prefer when it admits uncertainty.”; “Saying ‘I don’t know’ increases trust.”; “Transparency about limitations is important.” |
| Data privacy and confidentiality | Protection of personal information | 24 | “I worry about my personal data.”; “Protecting my information is very important.”; “Privacy strongly affects my trust.” |
|  | Secure data storage | 19 | “I want assurance that data is stored securely.”; “Security measures should be explained.”; “Secure systems increase confidence.” |
| Institutional endorsement and regulation | Endorsement by health authorities | 21 | “I trust it more if it is approved by health authorities.”; “Government endorsement increases credibility.”; “Official approval makes it safer.” |
|  | Professional oversight | 16 | “There should be medical supervision.”; “Doctors should be involved in development.”; “Professional oversight increases reliability.” |
| Risk of misleading or harmful advice | Incorrect advice | 22 | “Wrong advice could be harmful.”; “Incorrect information reduces trust immediately.”; “Accuracy is critical to avoid harm.” |
|  | Over-reliance on AI | 15 | “People should not rely on AI too much.”; “Over-dependence is risky.”; “AI should not replace professional judgement.” |
| Q3. Future role of AI | | | |
| Supportive and complementary role | Preliminary guidance only | 24 | “It should provide initial guidance only.”; “AI should support, not replace doctors.”; “It works best for preliminary advice.” |
|  | Referral to healthcare professionals | 22 | “It should encourage consulting a doctor.”; “Referral features are important.”; “It should guide users to professional care.” |
| Integration within institutional health services | University-provided tools | 20 | “Universities should provide official chatbots.”; “Student health services should include AI.”; “Institutional tools feel more trustworthy.” |
|  | Standardised platforms | 15 | “One standard platform would be helpful.”; “Consistency across services is important.”; “Standardisation improves reliability.” |
| Regulation and governance | Ethical frameworks | 18 | “Ethical guidelines are essential.”; “AI must follow ethical principles.”; “Ethics should guide implementation.” |
|  | Clear boundaries of use | 21 | “Clear limits should be set.”; “AI should not go beyond its role.”; “Boundaries prevent misuse.” |
| Education and digital health literacy | Improving health literacy | 17 | “It can educate students about health.”; “AI improves health awareness.”; “It helps students understand health information.” |
|  | Teaching critical evaluation | 13 | “It should teach users how to evaluate information.”; “Critical thinking is important.”; “Students should verify advice.” |
| Cautious and responsible expansion | Gradual implementation | 14 | “It should be introduced gradually.”; “Careful implementation is necessary.”; “Testing before full use is important.” |
|  | Continuous evaluation | 19 | “The system should be regularly evaluated.”; “Ongoing monitoring is essential.”; “Feedback should guide improvements.” |
